# Inhibition of Transketolase Improves the Prognosis of Colorectal Cancer

**DOI:** 10.3389/fmed.2022.837143

**Published:** 2022-02-23

**Authors:** Linhao Zhang, Zhiyin Huang, Qiuyu Cai, Chong Zhao, Yang Xiao, Xin Quan, Chengwei Tang, Jinhang Gao

**Affiliations:** ^1^Lab of Gastroenterology and Hepatology, West China Hospital, Sichuan University, Chengdu, China; ^2^Department of Gastroenterology, West China Hospital, Sichuan University, Chengdu, China

**Keywords:** colorectal cancer, transketolase, prognostic biomarker, survival highlights, Notch

## Abstract

Colorectal cancer (CRC) remains a heavy health burden worldwide. Transketolase (TKT) is a crucial enzyme in the non-oxidative phase of the Pentose Phosphate Pathway (PPP), and is up-regulated in multiple cancer types. However, the role of TKT in the prognosis of CRC remains unclear. We aimed to explore whether TKT expression is altered in CRC, how TKT is associated with the prognosis of CRC, and whether the regulation of TKT might have an impact on CRC. Differentially expressed genes (DEGs) were identified using bioinformatics analysis. TKT expression was examined in the human colon adenocarcinoma tissue microarray and xenografts. Cell viability, proliferation, migration, and apoptosis assays *in vitro* were applied to evaluate the protumoral effects of TKT on CRC. TKT was found to be a risk factor for the poor prognosis of CRC by bioinformatics analysis among the DEGs. TKT was significantly up-regulated in colon adenocarcinoma tissues compared with normal colon tissues in patients. Moreover, similar results were found in HCT116 and RKO human colon adenocarcinoma xenografts in nude mice. TKT expression was positively associated with advanced TNM stage, positive lymph nodes, and poor 5 or 10-year overall survival of CRC patients. *In vitro*, inhibition of TKT reduced cell viability, proliferation, and migration, and induced cell apoptosis. In addition, inhibition of TKT decreased the protein levels of NICD and Hes1. In conclusion, high TKT expression was associated with the poor prognosis of CRC patients. The protumoral effects of downregulating TKT may be realized by suppressing the Notch signaling pathway. TKT may be a new prognostic biomarker and therapeutic target for CRC.

## Highlights

- High TKT expression predicts poor prognosis of CRC patients.
- The protumoral effects of TKT are related to the Notch signaling pathway.
- TKT may be a new prognostic biomarker and therapeutic target for CRC.

## Introduction

Colorectal cancer (CRC) ranks second in mortality and third in incidence among all cancer types worldwide (1). The incidence of CRC increases steadily in many countries (2), and approximately one in five patients have metastatic disease at the time of presentation (3). Treatment of CRC usually consists of surgical removal of the tumor, chemotherapy and/or targeted therapy (4). However, there is still a lack of effective therapeutic targets due to tumor metastasis, recurrence and drug resistance (5–7). It is crucial to find suitable biomarkers and therapeutic targets for better prognosis evaluation and therapeutic interventions.

Previous studies have reported that many factors can influence the prognosis of CRC, including RAS mutational status, HER2 amplification, miRNAs and inflammatory markers (5, 8–10). However, the lack of targeted drugs for RAS mutations, resistance to anti-EGFR therapy, difficulty in detecting miRNAs and lack of further validation experiments for inflammatory markers restrict their clinical use (4, 11). Transketolase (TKT) is a crucial enzyme in the non-oxidative phase of the Pentose Phosphate Pathway (PPP) of glucose metabolism, and bridges the oxidative part of the PPP by NADPH production (12). TKT, which is expressed in all investigated organisms and most tumor tissues (13), enables cells to meet their anabolic demands under different conditions (14) and to convert glucose to ribose for nucleic acid synthesis (15). As an essential enzyme in metabolism, TKT promotes hepatocellular carcinoma *via* metabolic mechanisms, nuclear localization, and the EGFR pathway (13). Meanwhile, deficiency of TKT protected the liver from DNA damage (16). Moreover, high levels of TKT resulted in poor survival of breast cancer *via* the α-ketoglutarate signaling pathway (17). In CRC, TKT expression was predicted to be enhanced during cell cycle progression, and up-regulation of TKT was associated with invasive tumor cells (18, 19). However, few studies have focused on the relationship between TKT and the prognosis of CRC.

In this study, we demonstrated that high TKT expression was related to the poor prognosis of CRC. Inhibition of TKT reduced the malignancy of CRC, and was associated with downregulation of the Notch signaling pathway. The current study indicated that TKT might be a new prognostic biomarker and therapeutic target for CRC.

## Materials and Methods

### Identification of Differentially Expressed Genes (DEGs)

Four gene expression profiles (GSE8671, GSE32323, GSE24514, and GSE14333) were obtained from the Gene Expression Omnibus (GEO) database. Detailed information is listed in Supplementary Table 1. Briefly, GSE8671 included 32 colorectal adenoma tissues and 32 matched normal colorectal tissues. GSE32323 consisted of 17 CRC tissues and 17 normal colorectal tissues. GSE24514 included 34 colorectal tumor tissues and 15 normal colorectal tissues. GSE14333 included 140 CRC tissues of patients with disease-free survival longer than 50 months and 86 CRC tissues of patients with disease-free survival shorter than 50 months. The DEGs between different sample groups were identified by GEO2R (https://www.ncbi.nlm.nih.gov/geo/geo2r/). The cut-off criteria were log (FC)>1, *P*-value <0.01.

### Analysis of Key Genes Expressions

The bioinformatic analysis was done as previously reported (20). Gene expression profiling interactive analysis (GEPIA) (http://gepia.cancer-pku.cn/) is a collective web server for analyzing the RNA expression data of 9,736 tumors and 8,587 normal samples from the TCGA (The Cancer Genome Atlas) and the GTEx (Genotype-Tissue Expression) projects. Differential expression of key genes in CRC tissues and normal tissues was analyzed using the GEPIA database. Key genes related to the 5-year overall survival of CRC patients were also obtained from the GEPIA database. *P* <0.01 was regarded as the threshold.

### The TKT Mutations and TKT Associate Genes in CRC

The mutation status of TKT in human cancers was obtained from the TIMER2.0 database (http://timer.cistrome.org/) (21). The TKT associated genes were retrieved from the LinkedOmics database (http://linkedomics.org/) (22). Briefly, the colorectal adenocarcinoma (COAD, READ) was selected as the cancer type, and RNA-seq data from the HiSeq RNA platform were selected as the search dataset and target dataset. Colon adenocarcinoma containing 391 cases was chosen as the sample dataset to display the TKT associated genes, and the Pearson correlation test was selected as the statistical method. For the Gene Set Enrichment Analysis (GSEA), the Reactome pathway with a minimum number of genes of 3 and simulations of 500 was selected.

### Tissue Microarray (TMA)

Human colon adenocarcinoma TMA was provided by Shanghai Outdo Biotech (Shanghai, China). TMA samples contained 100 colon adenocarcinoma tissues collected from patients who underwent complete surgical resection of CRC between July 2005 and December 2010. Of these, 60 corresponding paracancerous tissues were also collected. Written informed consent forms were delivered to all the patients before participation, and ethical approval was obtained from the ethical committee of related hospitals.

### Cell Culture

The 3 human colon cancer cell lines were bought from the Procell Life Science and Technology Co. Ltd. (Wuhan, China). HCT116 cells were cultured with RPMI-1640 (HyClone, Logan, UT, USA) containing 10% fetal bovine serum (FBS, Gibco, Logan, UT, USA). RKO cells and Ls174T cells were cultured with Dulbecco's modified Eagle's medium (DMEM, HyClone) containing 10% FBS. Cells were incubated in a humidified atmosphere of at 37°C with 5% CO_2_ in air.

### Human Colon Cancer Xenografts

Twelve healthy male BAL b/c nude mice, weighing 18–22 g, were obtained from the Experimental Animal Center of Sichuan University (Chengdu, China). The mice were housed under a 12-h light/dark cycle, receiving food and water *ad libitum* at a constant temperature and humidity. Mice were randomly allocated to HCT116 and RKO groups, with 6 in each group. In brief, a total of 1 ×10^7^ HCT116 cells or RKO cells were subcutaneously injected into the left abdomen of 6 nude mice. Four weeks later, the tumors were aseptically resected, and the corresponding colon was also collected after the mice were sacrificed. The animal operations were approved by the Ethics Committees of Sichuan University and were conducted according to the regulations of Sichuan University.

### Hematoxylin and Eosin Staining (H&E)

TMA sections and mouse sections were routinely deparaffinized in xylene and rehydrated with a series of ethanol dilutions. H&E staining was operated according to the manufacturer's instructions (Solarbio, Beijing, China).

### Immunohistochemistry (IHC) Staining

TMA sections and mouse slides were routinely deparaffinized in xylene and rehydrated with a series of ethanol dilutions. Heat-induced antigen retrieval was performed in sodium citrate buffer (10 mM, pH = 6.0) for 30 mins. Then, the sections were first blocked by H_2_O_2_, and antigen blocking was performed with 10% goat serum afterward. Subsequently, all sections were incubated with rabbit anti-TKT (Proteintech, Wuhan, China) overnight at 4°C followed by incubation with horseradish peroxidase-conjugated secondary antibody kits (ZSGB Bio, Beijing, China) at 37°C for 30 mins. Finally, the sections were stained with diaminobenzidine tetrahydrochloride and counterstained with hematoxylin.

### IHC Scoring

The abovementioned TMA slides were assessed by two independent pathologists blinded to the pathological and clinical information. The expression of TKT in the tissues was scored semi-quantitatively, combining the positive rate and the intensity of the stained portion (staining index = positive × intensity score) based on previous studies (23–26). The positive percentage of the stained tumor cells was scored as follows: 0, no staining; 1, <20%; 2, 20–75%; and 3, > 75%. The intensity of the stained tumor cells was graded on the following scale: 0, negative; 1, weak; 2, moderate; and 3, strong staining. According to the staining index, a final calculating score of 0–4 was regarded as a low expression of TKT, and a final score of 5–9 was considered a high expression of TKT. The expression of TKT in paraffin-embedded sections from the mice was evaluated according to the integrated optical density (IOD) measured by Image-Pro Plus 6.0 software (Media Cybernetics, Rockville, MD, USA).

### Knock-Down of TKT With SiRNA

The TKT siRNA sequences (sense: GGC UGU GUC CAG UGC AGU AdTdT, anti-sense: UAC UGC ACU GGA CAC AGC CdTdT) were designed based on the cDNA sequence of TKT obtained from GenBank. HCT116 cells were transfected with TKT siRNA applying Lipofectamine (Invitrogen, Carlsbad, CA, USA) according to the manufacturer's instructions. Besides, a sequence (si-NC) was synthesized as a negative control.

### Cell Viability by CCK8 Assay

A CCK8 assay was used to evaluate the viability of the cells. HCT116 and LS174T cells were seeded into a 96-well plate. TKT inhibitor N3PT treatment (DMSO group, 5 μM N3PT group, 10 μM N3PT group) and siRNA transfection were performed. After 24 h of treatment with N3PT or 72 instructions (Dojindo, Kumamoto, Japan). The optical density of the cells was measured by a Thermo microplate reader (Thermo Fisher Scientific, Waltham, MA, USA) at 450 nm.

### Cell Proliferation by Immunofluorescent (IF) Staining of Ki-67

HCT116 and LS174T cells were seeded on glass chamber slides. Cells were treated with N3PT for 24 h or siRNA transfection for 72 h, and then fixed with 4% paraformaldehyde for 15 mins. After permeabilization with 0.2% Triton X-100 for 15 mins, antigen blocking was performed with 10% goat serum for 1 h at 37°C. The slides were then incubated with primary antibody against Ki-67 (Abcam, Cambridge, UK) overnight at 4°C followed by a goat anti-rabbit fluorescent secondary antibody (Abcam, Bristol, UK). The slides were counterstained with DAPI. An optical microscope (CX41, Olympus, Tokyo, Japan) equipped with a camera (DP72, Olympus) was applied to capture images of the slides.

### Cell Apoptosis by Annexin V/PI

Cell apoptosis was evaluated using Annexin V FITC-A/PI PE-A (BD Biosciences, Waltham, MA, USA) according to the manufacturer's instructions. After the corresponding treatments, cells and medium from all wells were collected into a 5 mL plastic tube. The tubes were centrifuged for 5 mins at 1,500 rpm and 4°C, and the supernatant was discarded afterward. Subsequently, 400 μL of 1 × binding buffer was added. Then, 5 μL Annexin V was first added to the cells, followed by 5 μL PI. Finally, flow cytometric analysis (Beckman Coulter, Brea, CA, USA) was immediately performed. Early apoptotic cells were labeled Annexin V positive and PI negative, whereas late apoptotic cells were recognized as positive for Annexin V and PI.

### Cell Migration by Wound-Healing Assay

After 90% confluence, cells were scratched by a 200 μL pipette tip. Then, the cells were softly washed with PBS for 3 times to remove the debris. Scratched cells were immediately captured by a microscope. Afterwards, cells were treated with si-TKT or N3PT and photographed after 24 and 48 h. Cell migration was calculated according to a previous study (27).

### Cell Migration by Transwell Assay

Transwell chambers (Corning, Corning, NY, USA) were applied to further calculate cell migration ability. Briefly, HCT116 cells treated with si-TKT or N3PT were added to the upper serum-free chambers. The corresponding lower chambers served as a chemo-attractant containing 10% FBS medium. After incubation at 37°C for 24 h (for N3PT) or 72 h (for si-TKT), cells that migrated onto the membrane surface were fixed with formaldehyde and then stained with hematoxylin for 30 mins. The number of cells that migrated to the lower compartment of the chamber was captured and counted by optical microscopy (CX41, Olympus) in five randomly selected views.

### Western Blot

The extraction of the whole proteins from the cultured cells was performed using RIPA buffer (Beyotime Biotechnology, Shanghai, China). The same amount of protein (30 μg) from all cell samples was analyzed by 10% SDS-PAGE and transferred onto PVDF membranes (Millipore, Billerica, MA, USA). The PVDF membranes were first blocked with 5% non-fat dry milk, followed by incubation with primary antibodies directed against GAPDH (1:5000, Abcam, Cambridge, UK), NICD (Abcam, Cambridge, UK), TKT (Proteintech) and Hes1 (SAB, College Park, MD, USA), respectively at 4°C overnight. After washing in TBST, the membranes were incubated with suitable secondary antibodies (Santa Cruz Biotechnology, TX, USA) for 2 h. The bands were visualized using an ECL detection kit (Santa Cruz Biotechnology, TX, USA). The intensity of the bands was determined by Quantity One software 4.6.2 (Bio-Rad, Hercules, CA, USA), and the expression levels of proteins were normalized to GAPDH. The results were displayed as fold changes compared to the control group.

### Statistical Analysis

All data were presented as the mean ± standard deviation and were analyzed using SPSS 19.0 software (SPSS, Chicago, IL, USA). Quantitative data were analyzed by the Student's *t*-test. Survival curves were generated using the Kaplan-Meier method and were compared using the log-rank test. The difference in clinical and pathological parameters between the low and high TKT groups was analyzed by the χ^2^ test. For multi-group comparisons, one-way ANOVA followed by SNK multiple comparison tests was implemented. *P* <0.05 was deemed statistically significant.

## Results

### Bioinformatics Analysis of TKT as a Prognostic Biomarker for CRC

**Figure 1:**
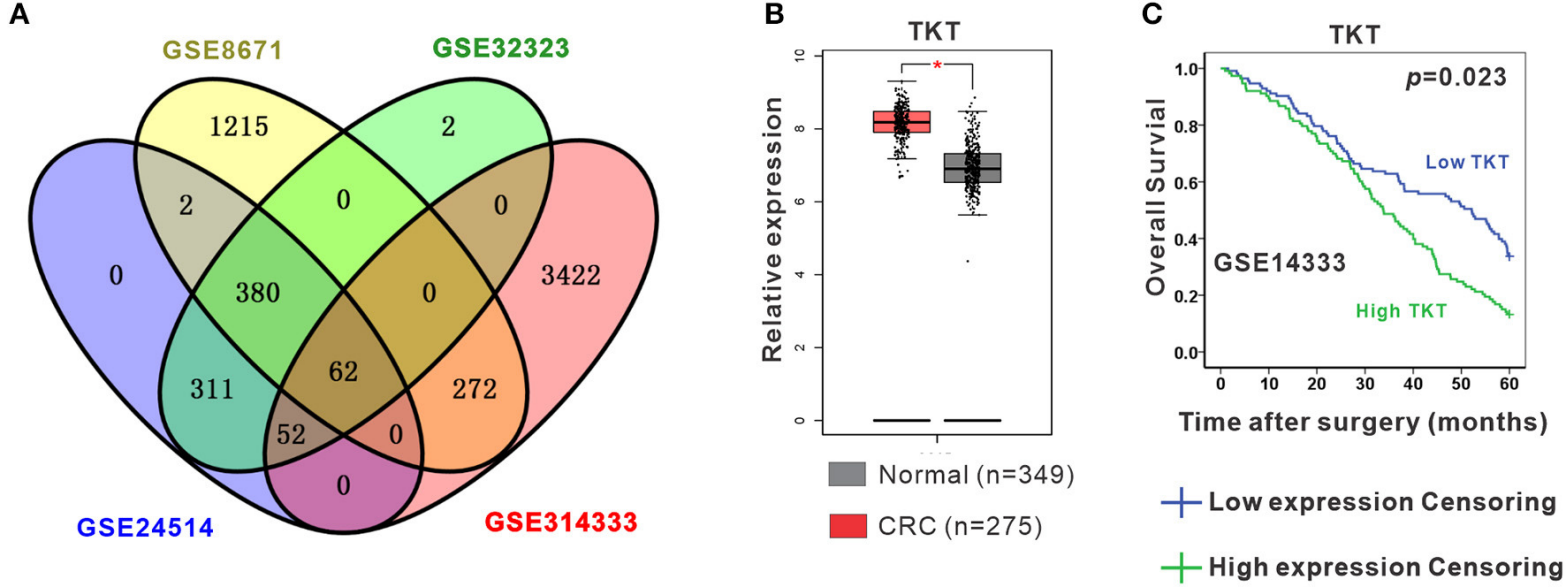
Identification of differentially expressed genes (DEGs). **(A)** Four datasets (GSE8671, GSE32323, GSE24514, GSE14333) were retrieved from the GEO database to identify the shared DEGs in CRC. Eventually, a total of 62 shared DEGs were identified using GEO2R; **(B)** TKT was significantly and highly expressed in CRC tissues compared to normal colon tissues in the GEPIA database; **(C)** High TKT expression in CRC was associated with poor 5-year survival in the GEPIA database. ^*^P <0.05 *vs*. normal colon tissues.

To identify the DEGs in CRC, 4 gene expression profiles (GSE8671, GSE32323, GSE24514, and GSE14333) were obtained from the Gene Expression Omnibus database (Supplementary Table 1). A total of 62 shared DEGs were identified in all four databases mentioned above using GEO2R (Figure 1A; Supplementary Table 2). Among the genes, 12 genes presented the most significant differential expression between CRC tissues and normal tissues using GEPIA analysis (Supplementary Figure 1; Supplementary Table 2). Finally, TKT and MYH11 were determined to be related to the survival period by GEPIA overall survival analysis (Figures 1B,C; Supplementary Figure 2). Compared to normal colon tissues, TKT was highly expressed in CRC tissues, and high TKT expression was associated with worse overall survival, which met our expectations (*P* = 0.023, Figures 1B,C). MYH11 expression was lower in CRC tissues than in normal colon tissues, whereas high MYH11 expression indicated poor prognosis in CRC patients (*P* = 0.024, Supplementary Figure 2). Therefore, we chose TKT as the target gene for our subsequent research.

### Up-Regulation of TKT in Colon Adenocarcinoma

**Figure 2:**
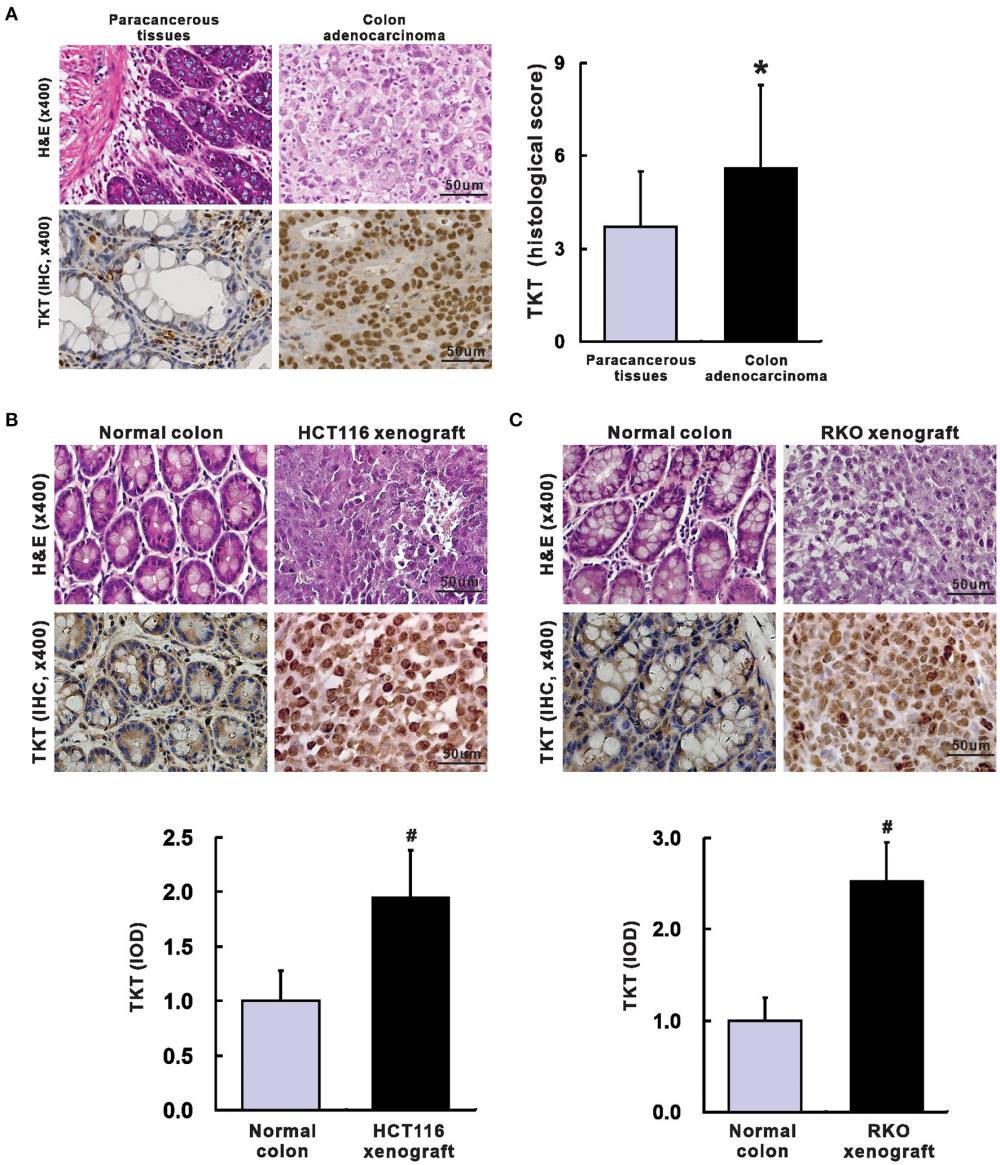
Up-regulation of TKT in colon adenocarcinoma. **(A)** Representative images for H&E (left up) and IHC of TKT (left lower) were shown, and the histological score of TKT for colon adenocarcinoma tissues (right) was significantly higher than that for the corresponding paracancerous tissues, *N* = 60 in the paracancerous tissues group, N = 100 in the colon adenocarcinoma tissues group; **(B)** Representative images for H&E (upper panel of images) and IHC of TKT (lower panel of images) were shown, and the histological score of TKT for HCT 116 xenografts, whose expression was significantly higher than that for the corresponding normal colon tissues; **(C)** Representative images for H&E (upper panel of images) and IHC of TKT (lower panel of images) were shown, and the histological score of TKT for RKO xenografts, whose expression was significantly higher than that for the corresponding normal colon tissues, *N* = 6/group. *P <0.05 *vs*. paracancerous tissues; ^#^*P* <0.05 *vs*. normal colon tissues in mice.

Next, a TMA containing 100 colon adenocarcinoma tissues and 60 corresponding paracancerous tissues was applied to verify whether TKT could serve as a prognostic biomarker for CRC. The clinical and pathological characteristics of the 100 patients recruited in this study were shown in Table 1. In the TMA sections of patients with colon adenocarcinoma, H&E staining showed typical colon adenocarcinoma histology, while the corresponding paracancerous tissues presented typical colon structures with columnar epithelial cells in a circular arrangement pattern interspersed by goblet cells (Figure 2A). Then, IHC staining for TKT was performed in the TMA. For colon adenocarcinoma tissues, positive staining was mainly distributed in the nucleus of the tumor cells. However, paracancerous tissues rarely expressed TKT (Figure 2A). There was a significant difference between the staining index of colon adenocarcinoma tissues and the corresponding paracancerous tissues (5.6 ± 2.7 *vs*. 3.7 ± 1.8; *P* <0.05, Figure 2A).

**Table 1:** Relationships between TKT expression and clinicopathological characteristics in 100 colon adenocarcinoma cases.

**Characteristics**	**Number of total cases**	**TKT expression (%)**		***P*-value**
		**Low**	**High**	
Total	100	39	61	
Average years	62.4 ± 11.6	59.9 ± 12.2	64.0 ± 11.1	
<65	54 (54.0)	25 (64.1)	29 (47.5)	0.150
≥65	46 (46.0)	14 (35.9)	32 (52.5)	
Gender				
Male	58 (58.0)	25 (64.1)	33 (54.1)	0.407
Female	42 (42.0)	14 (35.9)	28 (45.9)	
Histologic grade				
I–II	78 (78.8)	27 (69.2)	51 (83.6)	0.136
II–III	22 (21.2)	12 (30.8)	10 (16.4)	
TNM stage				
I–II	61 (61.0)	29 (74.4)	32 (52.5)	0.036
III–IV	39 (39.0)	10 (25.6)	29 (47.5)	
Location (cm)				0.837
Left colon	42 (42.0)	17 (43.6)	25 (41.0)	
Right colon	58 (58.0)	22 (56.4)	36 (59.0)	
Infiltration degree				
Adventitia	70 (70.0)	29 (74.4)	41 (67.2)	0.507
Serosa/muscular/mucosa	30 (30.0)	10 (25.6)	20 (32.8)	
Pathological morphology				
Infiltrate /ulcer type	75 (75.0)	28 (71.8)	47 (77.0)	0.638
Protrude/ basin type	25 (25.0)	11 (28.2)	14 (23.0)	
Distant metastasis and Vascular invasion	18 (18.0)	6 (15.4)	12 (19.7)	0.790
Positive lymph nodes	38 (38.0)	9 (23.1)	29 (47.5)	0.020

We also verified TKT expression in HCT116 and RKO human colon adenocarcinoma xenografts in nude mice. Mice were sacrificed 4 weeks after tumor cell injection. There is no metastasis in all the mice. The tumor weight was 0.78 ± 0.15 g in HCT116 xenografts and 0.65 ± 0.13 g in RKO xenografts. H&E staining of HCT116 and RKO xenografts showed typical colon adenocarcinoma histological changes (Figures 2B,C). Moreover, HCT116 and RKO xenografts presented stronger expression of TKT than the corresponding normal colon tissues of the nude mice as evaluated by IOD (HCT116, 1.95 ± 0.43 *vs*. 1.00 ± 0.28, *P* <0.05, Figure 2B; RKO, 2.52 ± 0.43 *vs*. 1.00 ± 0.25, *P* <0.05, Figure 2C). In summary, TKT is up-regulated in colon adenocarcinoma.

### High TKT Expression Predicted Poor Prognosis in Human Colon Adenocarcinoma

**Figure 3:**
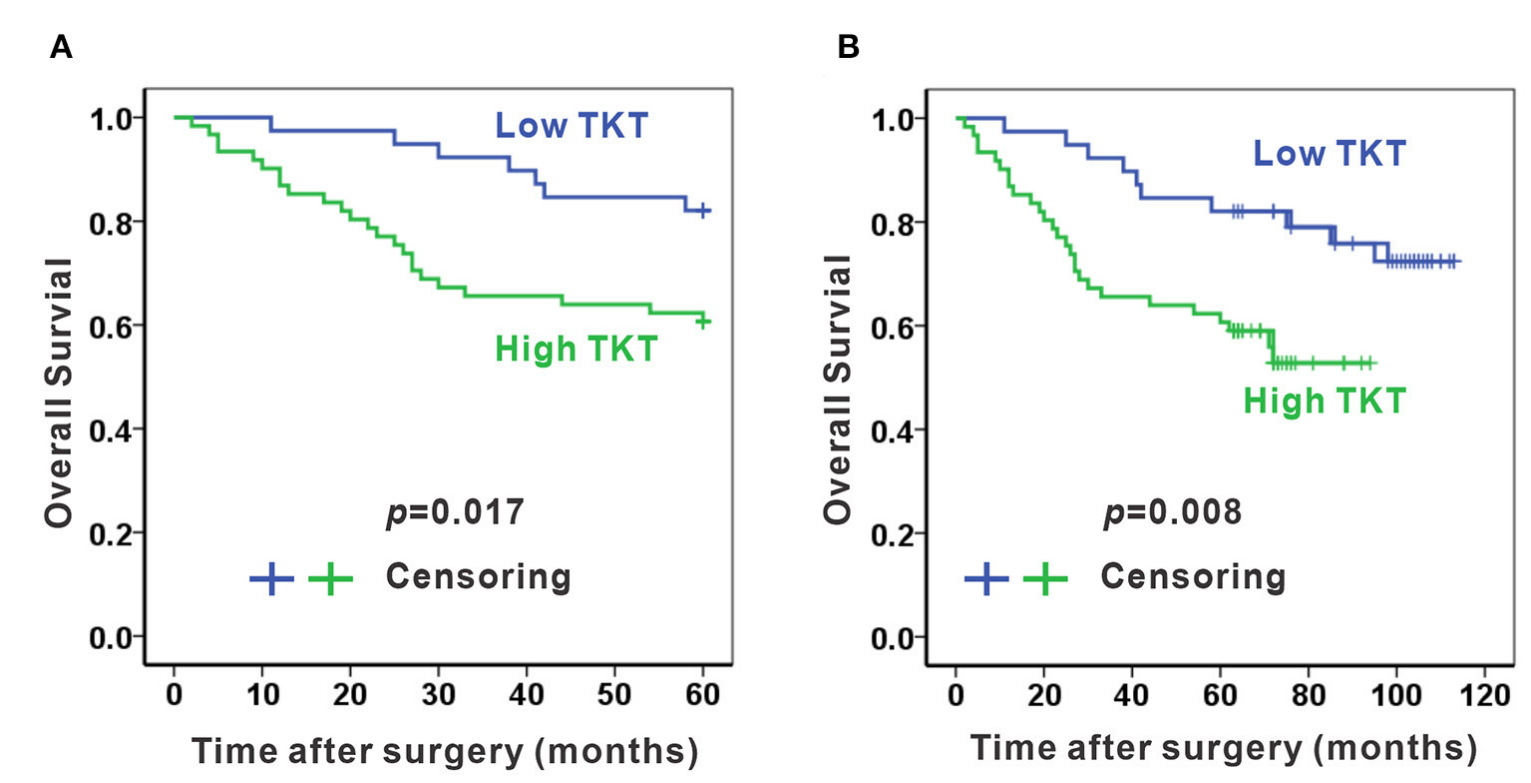
High TKT expression predicts poor 5-year and 10-year survival. High TKT expression was associated with lower 5-year overall survival **(A)** and lower 10-year overall survival **(B)**.

As TKT was up-regulated in colon adenocarcinoma, we evaluated the correlation of TKT expression and prognosis in human colon adenocarcinoma. Patients from the TMA were divided into 2 subgroups according to the staining index of TKT (Table 1). The low TKT group (staining index 0–4) contained 39 cases, and the high TKT group (staining index 5–9) included the other 61 cases. As for the TNM stage, 29 cases (74.4%) in the low TKT group were in stage I–II, and the remaining 10 cases (25.6%) were stage III–IV. However, 32 cases (52.2%) in the high TKT group were in stage I–II, and the remaining 29 cases (47.5%) were in stage III–IV. These results indicated that high TKT expression was associated with advanced TNM stages (*P* = 0.036, Table 1). Moreover, 9 cases (23.1%) were diagnosed with positive lymph nodes in the low TKT group. Conversely, 29 cases (47.5%) in the high TKT group were diagnosed with positive lymph nodes. Therefore, high expression of TKT might also be relevant to lymphatic metastasis (*P* = 0.020, Table 1). However, there was no significant correlation between the expression of TKT and other clinicopathological factors, such as age, gender, histologic grade, tumor location, infiltration degree, pathological morphology, distant metastasis and vascular invasion (*P* > 0.05, Table 1). Furthermore, the overall 5-year survival was shorter in the high TKT group than in the low TKT group (*P* = 0.017, Figure 3A). Consistently, high TKT expression was also significantly correlated with shorter 10-year survival (*P* = 0.008, Figure 3B). In summary, high TKT expression predicts poor prognosis in human colon adenocarcinoma.

### Inhibition of TKT Reduced Cell Viability and Proliferation, and Induced Cell Apoptosis

The above data indicate that TKT is up-regulated in colon adenocarcinoma, and that high TKT expression predicts poor prognosis in human colon adenocarcinoma. By bioinformatic analysis in TIMER2.0, we found that TKT is a low-frequency mutated gene (1.4%) in human colon adenocarcinoma (Supplementary Figure 3A). Then, the TKT-associated genes were retrieved from the LinkedOmics database. This result indicated that a number of genes were positively or negatively correlated with TKT in CRC (Supplementary Figures 3B, 4A,B). Moreover, by using GSEA and Reactome, we verified that TKT was involved in the metabolism of amino acids and derivatives and cell proliferation (S phase, G1/S transition, mitotic G1-GS/S phase, cell cycle) signaling pathways (Supplementary Figures 3C,D, 4C–F).

**Figure 4:**
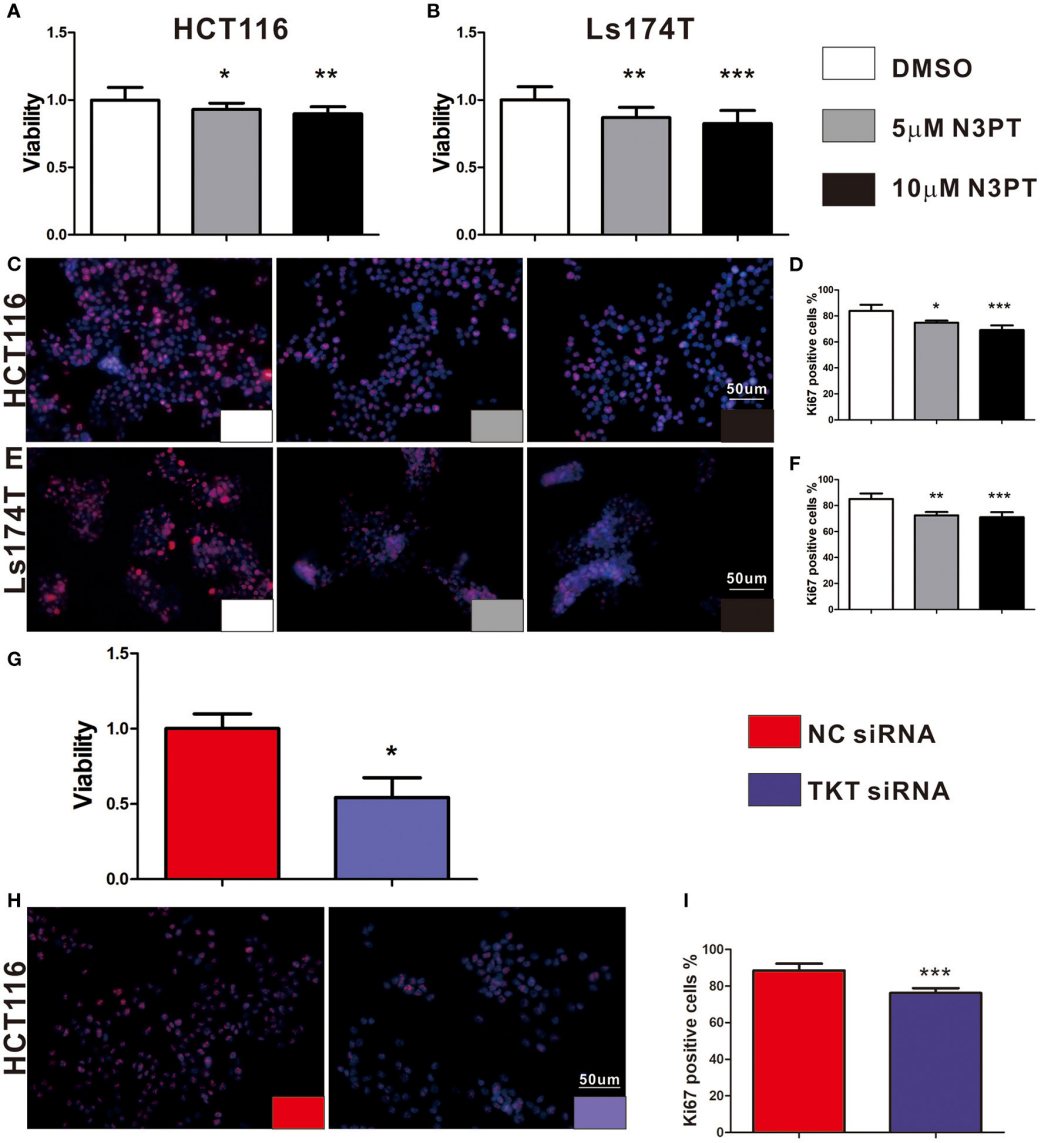
Inhibition of TKT reduces cell viability and proliferation. **(A–F)** HCT116 or Ls174T cells were treated with DMSO or the TKT inhibitor N3PT (5 or 10 μM) for 24 h. **(A,B)** Cell viability was quantified by CCK8 assay. The viability of HCT116 cells **(A)** and Ls174T cells **(B)** was reduced after treatment with 5 μM and 10 μM N3PT respectively. **(B–F)** Cell proliferation was determined by IF of Ki67 and quantified by Ki67 positive cells in each field. Fewer Ki-67-positive HCT116 cells **(C,D)** and Ls174T cells **(E,F)** were detected after N3PT treatment. **(G–I)** HCT116 cells were transfected with TKT siRNA or negative control (NC siRNA) for 72 h. **(G)** Cell viability was quantified by CCK8 assay. The viability of HCT116 cells was decreased after transfection with TKT siRNA. **(H,I)** Cell proliferation was determined by IF of Ki67 and quantified by Ki67 positive cells in each field. Fewer Ki-67-positive HCT116 cells were detected after transfection with TKT siRNA. *N* = 3/group, *P <0.05, **P <0.01, ***P <0.001 *vs*. the control group.

**Figure 5:**
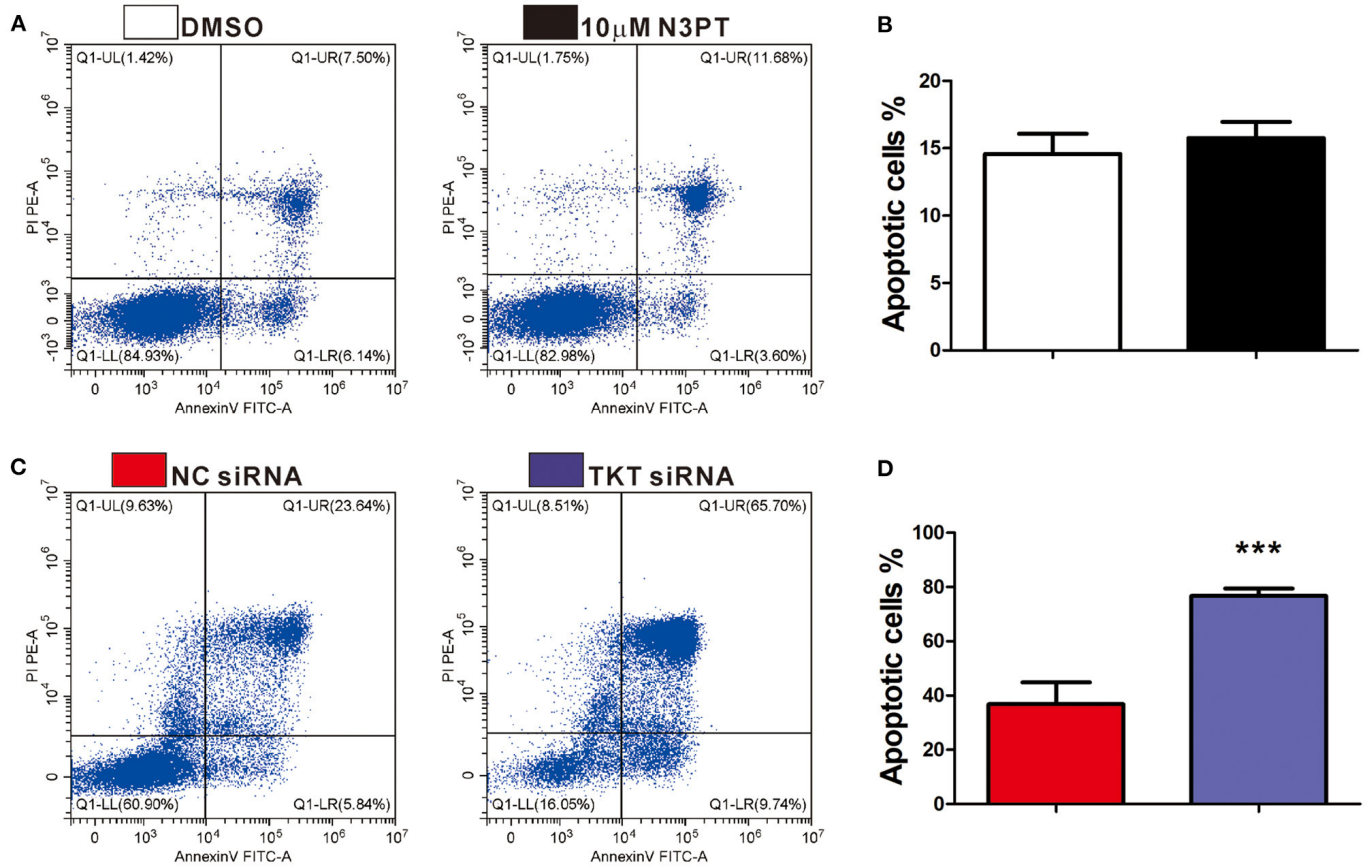
Inhibition of TKT induces cell apoptosis. **(A,B)** HCT116 cells were treated with DMSO or the TKT inhibitor N3PT (10 μM) for 24 h. Cell apoptosis was assayed by co-staining with Annexin V/PI. There is no significant difference in the apoptotic cell ratio between the 10 uM N3PT treated group and the control group. **(C,D)** HCT116 cells were transfected with TKT siRNA or negative control (NC siRNA) for 72 h. Cell apoptosis was assayed by co-staining with Annexin V/PI. The number of both early apoptotic and late apoptotic cells was significantly increased in the TKT siRNA group **(C)**. The apoptotic cell ratio was also significantly increased in the TKT siRNA group compared with the NC siRNA group **(D)**. Apoptotic cells are presented as the mean ± SD of three independent experiments (lower left: live cells, lower right: early apoptotic cells, upper right: late apoptotic cells, upper left: dead cells). *N* = 3/group, ^***^P <0.001 *vs*. the control group.

As cell proliferation was enriched in the TKT-related signaling pathway, we next investigated the anti-tumoral effects of TKT inhibition on HCT116 and Ls174T cells. The cell proliferation of HCT116 cells determined by CCK8 and IF of Ki-67 showed a concentration-dependent decrease by the TKT inhibitor N3PT when compared to the control group (*P* <0.05, Figures 4A,C,D). Similar results were also obtained in Ls174T cells treated with N3PT (*P* <0.05, Figures 4B,E,F). To show the direct effect of TKT on cell proliferation, HCT116 cells were selected as the *in vitro* cell model for siRNA transfection. Significantly lower viability was shown in HCT116 cells transfected with TKT siRNA compared with cells transfected with NC siRNA (*P* <0.05, Figure 4G). Consistently, compared with the NC siRNA group, cell proliferation quantified by IF of Ki-67 was significantly decreased in the TKT siRNA group (*P* <0.001, Figures 4H,I). Moreover, there was no significant difference in the apoptotic cell ratio between the 10 μM N3PT treated and control groups (Figures 5A,B). However, the apoptotic cell ratio was significantly increased in TKT siRNA transfected cells compared with NC siRNA-transfected cells (*P* <0.001, Figures 5C,D).

### Inhibition of TKT Suppressed Cell Migration

**Figure 6:**
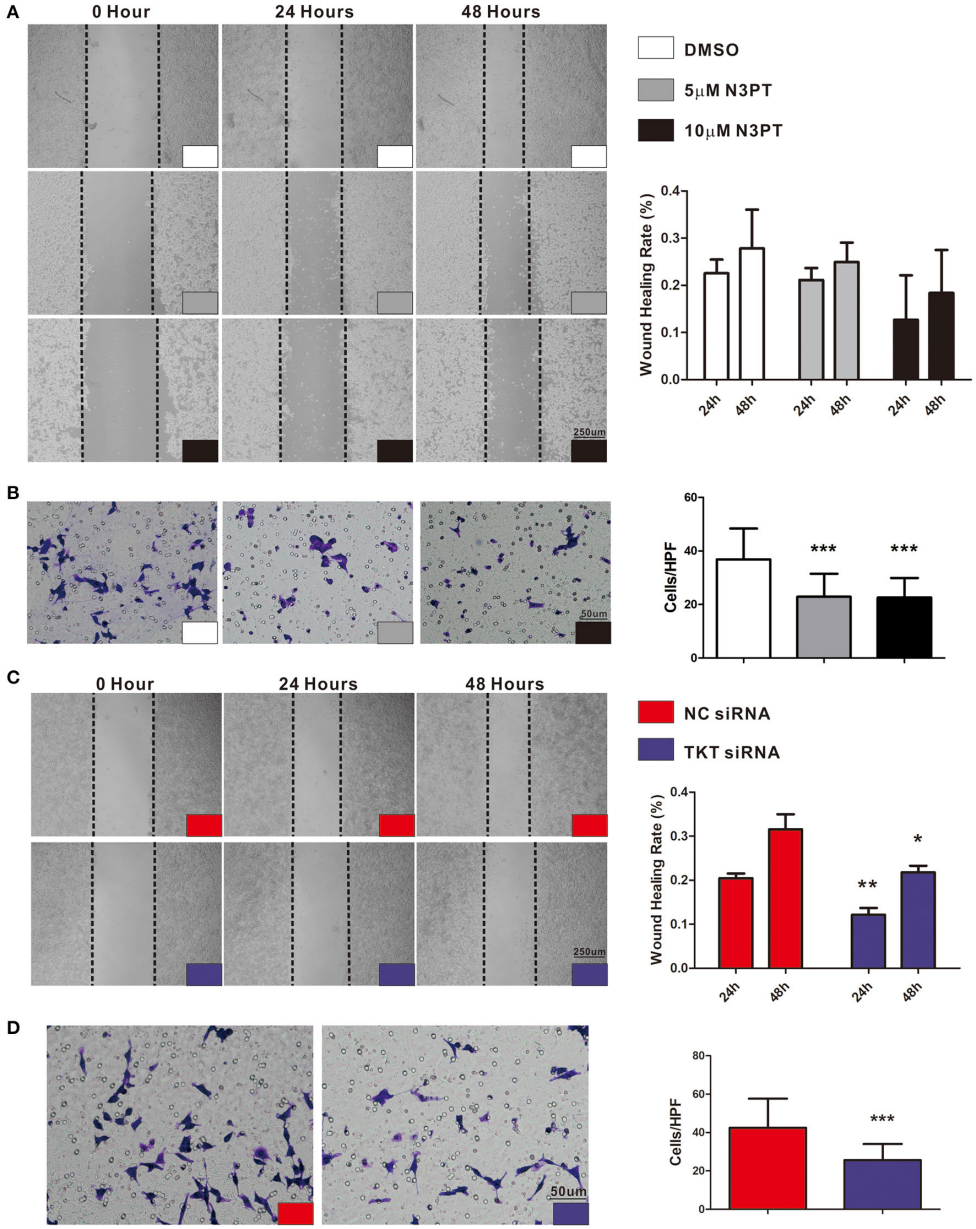
Inhibition of TKT suppresses cell migration. **(A,B)** HCT116 cells were treated with DMSO or the TKT inhibitor N3PT (5 μM or 10 μM), and cell migration was assayed by wound healing assay **(A)** or Transwell assay **(B)**. The wound-healing assays showed no significant difference in the migration rate among the 5 μM N3PT, 10 μM N3PT and control groups. However, the migration rate was significantly decreased in the 5 μM N3PT and 10 μM N3PT groups in a Transwell assay. **(C,D)** HCT116 cells were transfected with TKT siRNA or negative control (NC siRNA), and cell migration was assayed by wound healing assay **(C)** or Transwell assay **(D)**. TKT siRNA transfected cells presented a significantly lower wound healing rate **(C)** and migration ability **(D)** than the NC siRNA-transfected cells. *N* = 3/group, *P <0.05, **P <0.01, ***P <0.001 *vs*. the control group.

As tumor cell migration is a pivotal determinant of tumor metastasis, wound-healing assays and transwell chamber assays were utilized to evaluate the migration ability of cells with different treatments. Compared with control cells, cell migration was not significantly delayed after 24 and 48 h in either 5 μM or 10 μM N3PT treated HCT116 cells assessed by wound-healing assay (Figure 6A). Nevertheless, the migratory ability was significantly decreased in both 5 μM and 10 μM N3PT-treated HCT116 cells evaluated by Transwell assay (*P* <0.001, Figure 6B). For HCT116 cells transfected with TKT siRNA, cell recolonization into the wound area was significantly delayed compared with control cells after 24 and 48 h (*P* <0.05, Figure 6C). Similarly, the migratory ability determined by the Transwell assay was also significantly suppressed in the TKT siRNA-transfected cells (*P* <0.001, Figure 6D).

### Inhibition of TKT Was Associated With Downregulation of Notch Signaling

**Figure 7:**
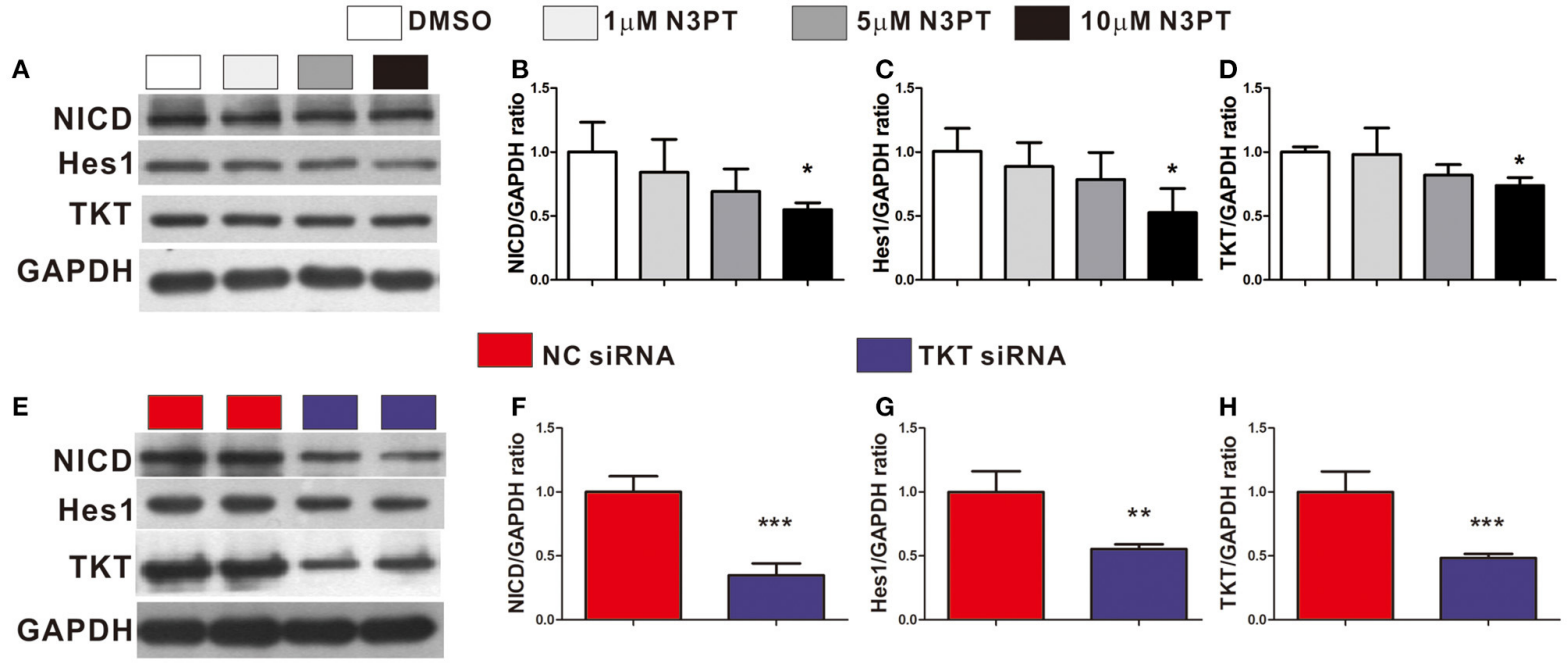
Inhibition of TKT blocks Notch signaling pathway. **(A–D)** HCT116 cells were treated with DMSO or the TKT inhibitor N3PT (1, 5, or 10 μM) for 24 h. Then the protein levels in cell lysates were quantified by Western blot **(A)**. The quantized data indicated that 10 μM N3PT treatment reduced the protein levels of NICD **(B)**, Hes1 **(C)**, and TKT **(D)** compared with the control. However, lower concentrations of N3PT had no significant effect on the protein levels of NICD, Hes1, and TKT **(A–D)**. **(E,F)** HCT116 cells were transfected with TKT siRNA or negative control (NC siRNA) for 72 h. Then the protein levels in cell lysates were quantified by Western blot **(E)**. Cells transfected with TKT siRNA presented lower protein levels of NICD **(F)**, Hes1 **(G)**, and TKT **(H)** than NC siRNA transfected cells. *N* = 3/group, *P <0.05, **P <0.01, ***P <0.001 vs. the control group.

The Notch signaling pathway is known to contribute to the tumor metastasis of CRC (28). In the Notch signaling pathway, Notch is a transmembrane receptor, NICD is its intracellular domain, and Hes1 is one of the downstream target genes (29). Notch and Hes1 were enriched in the bioinformatics analysis (Supplementary Table 2). Moreover, Hes1 and Notch were positively correlated with TKT expression by bioinformatics analysis (Hes1: R = 0.30; Notch: R = 0.39, Supplementary Figure 5). We next investigated the effects of TKT on the Notch signaling pathway. NICD, Hes1, and TKT proteins were significantly lower in the cells treated with 10 μM N3PT than in the control cells (*P* <0.05, Figures 7A–D). However, no significant decrease in the protein level was found when cells were treated with 1μM N3PT and 5μM N3PT (Figures 7A–D). Moreover, the reduction in NICD, Hes1 and TKT proteins was confirmed in the TKT siRNA transfected cells (*P* <0.05, Figures 7E–H). These results indicate that the inhibition of TKT is associated with the downregulation of the Notch signaling pathway.

## Discussion

**Figure 8:**
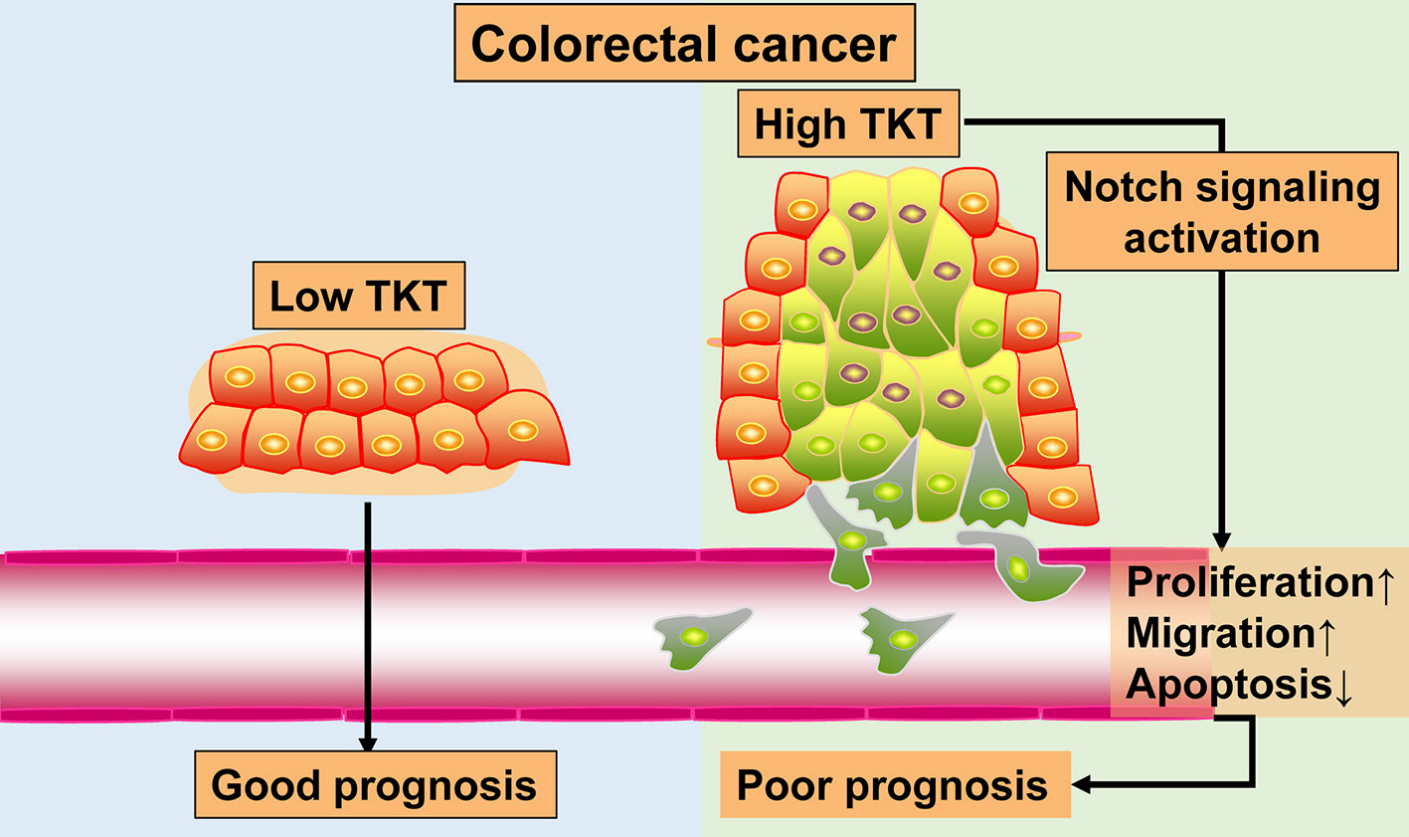
Schematic diagram.

Colorectal cancer remains a heavy health burden worldwide (30, 31), and the existing biomarkers and targets have limitations in the clinical treatment of CRC (4). The exploration of novel biomarkers and therapeutic targets is urgently needed to improve CRC therapy. In this study, by bioinformatics analysis of 4 gene expression profiles, TKT was found to be a prognostic biomarker for CRC. Furthermore, with TMA sections of CRC, we confirmed that high TKT expression was associated with advanced TNM stages and more positive lymph nodes, and predicted poor prognosis in patients. Besides, in the *in vitro* study, inhibition of TKT reduced cell viability and proliferation, induced cell apoptosis, and suppressed cell migration. These effects might be the result of downregulation of Notch signaling by TKT inhibition. The results of this study are summarized in a schematic diagram (Figure 8). These novel observations shed light on TKT as a new prognostic biomarker and therapeutic target for CRC. Moreover, effective therapy by inhibiting TKT should be developed for CRC patients.

Cancer cells use glycolysis over oxidative phosphorylation to produce energy even under aerobic conditions. The PPP is an essential metabolic pathway connected with glycolysis, and TKT is the major reversible enzyme in the non-oxidative branch of the PPP (12). Additionally, most nucleic acids of tumor cells come from ribose generated in the non-oxidative PPP branch (32). Thus, the up-regulated level of TKT might increase the ribose and energy needed for DNA and RNA synthesis. Previous studies (33, 34) showed that TKT was increased in cervical cancer and pancreatic cancer, and high TKT expression enhanced the non-oxidative PPP activity, providing raw materials for nucleic acid synthesis in tumor cells to meet the needs for growth and proliferation. However, the expression of TKT in CRC remains unknown. Our results showed that TKT was up-regulated in CRC, and a higher proportion of patients with high TKT expression had poor prognosis. In contrast, inhibition of TKT reduces tumor malignancy in CRC. These findings advance our understanding of TKT as a prognostic biomarker and therapeutic target for CRC. However, *in vivo* experiments are needed to validate whether high TKT expression is associated with lymphatic metastasis, advanced TNM stage, and poor survival.

The development and progression of CRC are closely related to the oxidative stress caused by the excessive production of reactive oxygen species (ROS) (35). TKT is an important enzyme that connects the PPP with glycolysis and induces ROS accumulation (36). In this study, CRC patients with high TKT expression more commonly presented with poor prognosis. These results may be explained by the high TKT level inducing more ROS damage to the colon. Consistently, non-oxidative PPP increased much more significantly than oxidative PPP due to the increased TKT activity in metastatic renal cancer (37). In addition, TKT might promote the development of HCC cells in non-metabolic manner, and nuclear localization of TKT predicted a poor prognosis for patients (13). Together with our results, these observations further validate that non-oxidative PPP catalyzed by TKT may play a pivotal role in the progression of CRC.

The Notch signaling pathway is known to be involved in the tumor metastasis of CRC (28). Notch is a transmembrane receptor, NICD is its intracellular domain, and Hes1 is one of the downstream target genes (29). In this study, inhibition of TKT either by N3PT or siRNA decreased the protein levels of NICD and Hes1. The overexpression of NICD and Hes1 could increase cell proliferation and decrease cell differentiation (38). We presume that inhibition of TKT might reduce tumor malignancy by blocking the Notch signaling pathway. Besides, many studies have reported that ROS induce the activation of Notch signaling, increasing the malignancy of cancer cells (39–41). Presumably, TKT may exert a pro-tumor effect by activating the Notch signaling pathway through ROS. However, further experiments are needed to verify the underlying molecular mechanism.

Taken together, CRC patients with high TKT expression more commonly presented with advanced TNM stage, positive lymph nodes and poor survival. Inhibition of TKT suppresses the proliferation and migration of CRC cells and induces cell apoptosis, which is associated with downregulation of the Notch signaling pathway. The current study indicates that TKT may serve as a new prognostic biomarker and therapeutic target for CRC.

## Data Availability Statement

The datasets presented in this study can be found in online repositories. The names of the repository/repositories and accession number(s) can be found in the article/Supplementary Material.

## Ethics Statement

Written informed consent forms were delivered to all the patients before participation, and ethical approval was obtained from the Ethical Committee of West China Hospital. The patients/participants provided their written informed consent to participate in this study. The animal study and operations were reviewed and approved by the Ethics Committees of Sichuan University and were conducted according to the regulations of Sichuan University.

## Author Contributions

JG and CT conceived and supervised the study. LZ, ZH, QC, CZ, YX, and XQ performed experiments. ZH and LZ analyzed the data. LZ, ZH, QC, and JG wrote the manuscript with input from all authors. All authors contributed to the article and approved the submitted version.

## Funding

This work was supported by the National Natural Science Fund of China (82170623, 82170625, U1702281, 81873584, 82000613, and 82000574), National Key R&D Program of China (2017YFA0205404), Sichuan Science and Technology Program (2020YJ0083 and 2021YFS0147), the 135 projects for disciplines of excellence (ZYGD18004) and Post-Doctor Research Project (2019HXBH013) of West China Hospital, Sichuan University, and China Postdoctoral Science Foundation Grant (2019M653436), and Science & Technology bureau of Chengdu (2017-CY02-00023-GX).

## Conflict of Interest

The authors declare that the research was conducted in the absence of any commercial or financial relationships that could be construed as a potential conflict of interest.

## Publisher's Note

All claims expressed in this article are solely those of the authors and do not necessarily represent those of their affiliated organizations, or those of the publisher, the editors and the reviewers. Any product that may be evaluated in this article, or claim that may be made by its manufacturer, is not guaranteed or endorsed by the publisher.
