# Supplementary material for: Inhibition of Transketolase Improves the Prognosis of Colorectal Cancer
Source: Front Med (Lausanne). 2022 Feb 23;9:837143. doi: 10.3389/fmed.2022.837143 (PMC8905541; doi:10.3389/fmed.2022.837143)
Supplement: Supplementary file 1 [file Data_Sheet_1.pdf]

Supplementary information

Supplementary Table S1 Characterize of 4 GEO datasets

Dataset	Tissue type	Platform	Normal	Tumor
GSE8671	colorectal adenoma	GPL570	32	32
GSE32323	CRC cancer	GPL570	17	17
GSE24514	Colorectal tumor	GPL96	15	34
GSE14333	CRC cancer	GPL570	86 (DFS>50)	140(DFS<50)

DFS: disease free survival

Supplementary Table S2 Target gene selection from DEGs among 4 GEO datasets

DEGs	Official gene name
**DEGs by overlap 4 datasets (62)**	MYH11, ZG16, GUCA2B, SORBS1, TAGLN, SUPT20H, TNS1, PYY, CALD1, IGH, CDH3, KIAA0101, PADI2, CDHR2, ENTPD5, MET, SLC12A2, TACSTD2, CXCL14, LRRC19, SMPDL3A, NEBL, CA12, PBLD, FNBP1, HES1, NOTH2, KLK10, EDN3, SLC7A5, TNXB, NT5DC2, SLCO4A1, TSPAN7, ANK2, HHLA2, ITM2C, CENPU, HOXB6, CD44, PRKCB, AKAP12, BIK, FMO5, CCL19, POU2AF1, TKT, CKAP2, SLC7A11, WWTR1, CHI3L1, IGHM, CENPN, ETS2, MYO1A, LGALS2, CD79A, PMP22, TRANK1, RIPK2, PTP4A1, FABP4
**Selected DEGs by expression (12)**	MET, TKT , TNS1, TAGLN, PYY, SLC7A11, CD44, ANK2, CALD1, CDH3, SORBS1, MYH11
**Selected DEGs by survival (2)**	TKT, MYH11

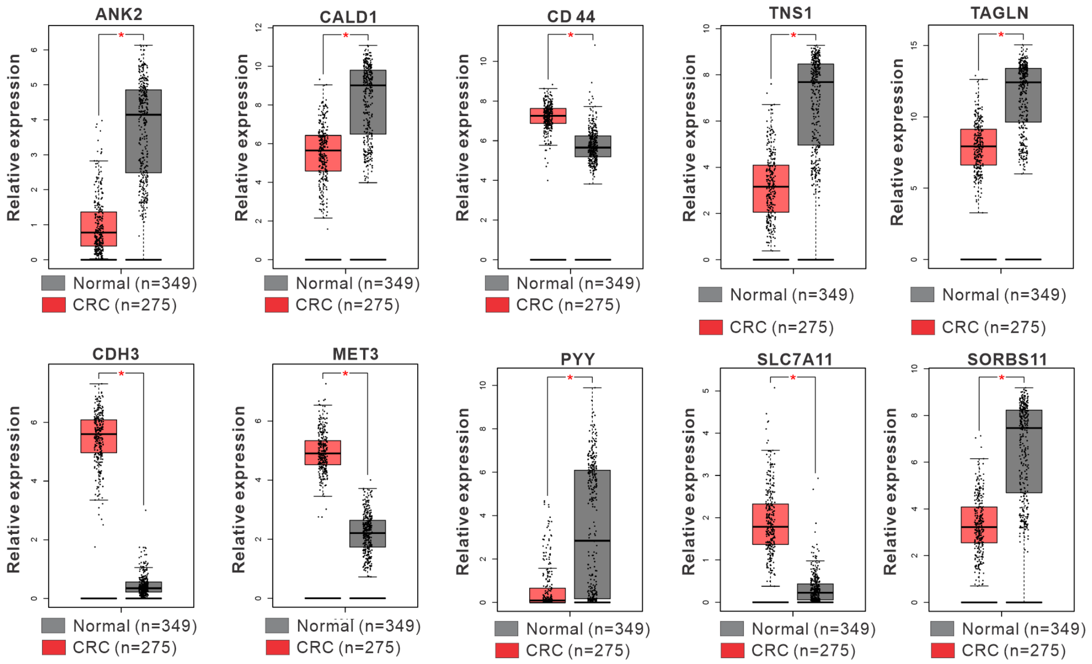

Supplementary Figure S1

Selected DEGs and their relative expressions in colon adenocarcinoma tissues and normal colon tissues

Relative expression of ANK2, CALD1, CD44, TNS1, TAGLN, CDH3, MET3, PYY, SLC7A11, SORBS11 in adenocarcinoma tissues and normal colon tissues. *P< 0.05 *vs*. normal colon tissues.

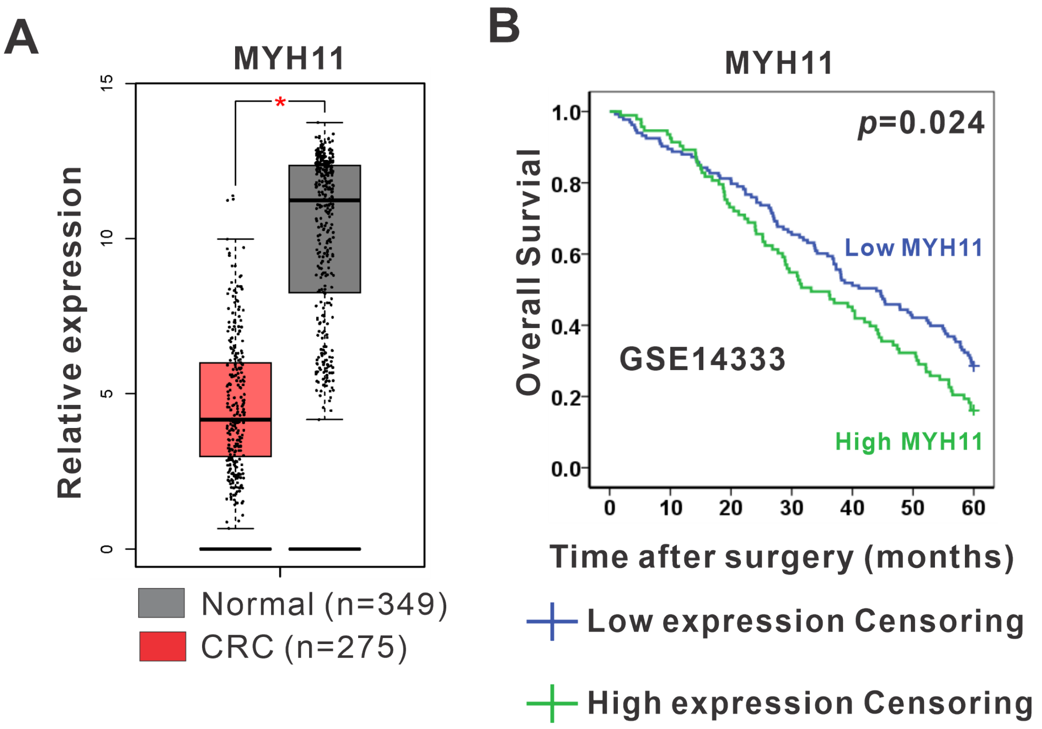

Supplementary Figure S2

High MYH11 expression in CRC associates with poor 5-year survival in the GEPIA database

A: MYH11 was lowly expressed in CRC tissues compared to normal colon tissues in the GEPIA database;

B: High MYH11 expression in CRC associates with poor 5-year survival in the GEPIA database. *P< 0.05 vs. normal colon tissues.

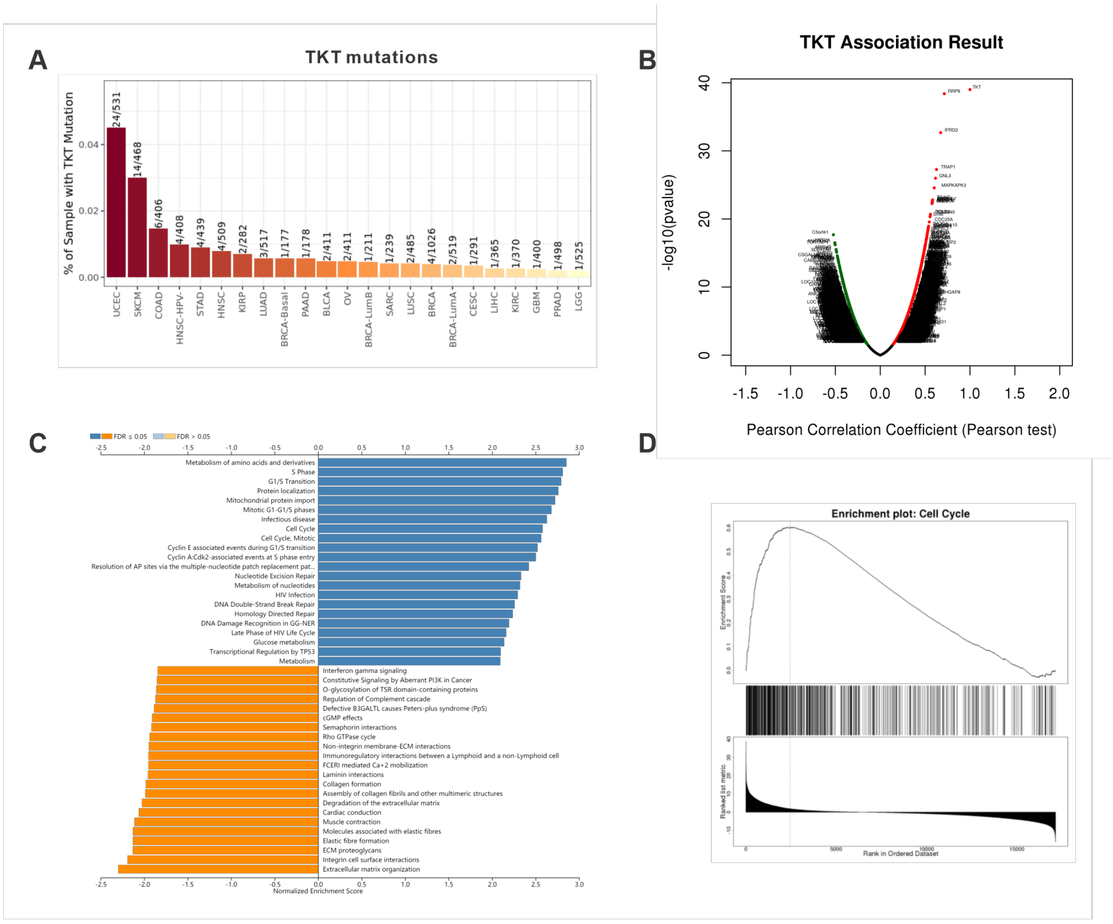

Supplementary Figure 3. The TKT mutations and TKT associate genes in CRC

A: The mutation status of TKT in human cancers was obtained from the TIMER2.0 database. TKT is a low-frequency mutated gene (1.4%) in human colon adenocarcinoma;

B-D: The TKT associated genes were retrieved from the LinkedOmics database. The Pearson correlation analysis (B), the bar chart of GSEA Recatome analysis (C), and the enrichment plot of the cell cycle (D) were shown. These results indicated that a number of genes were positively or negatively correlated with TKT in CRC. Moreover, TKT has been involved in the metabolism of amino acids and derivatives and cell proliferation (S phase, G1/S transition, mitotic G1-GS/S phase, cell cycle) signaling pathways.

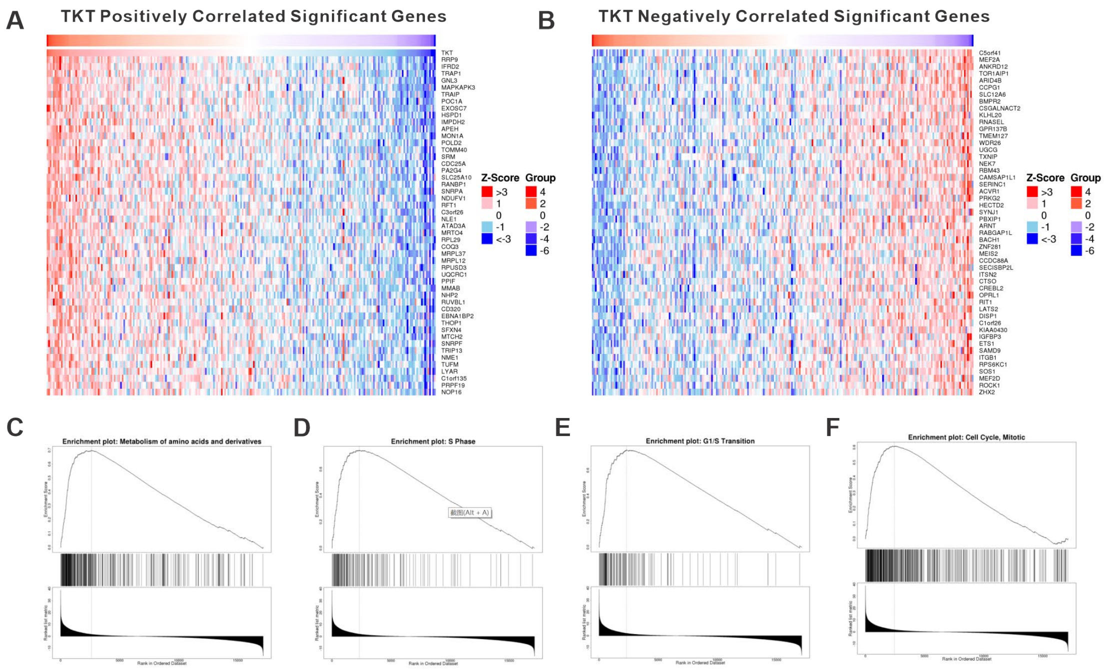

Supplementary Figure S4

TKT associate genes and enrichment signaling pathway in CRC

A-B: The TKT associate genes were retrieved from the LinkedOmics database. The heatmap of TKT positively correlated significant genes (A), and TKT negatively correlated significant genes (B).

C-F: The enrichment plot of the Reactome enriched signaling pathway was shown. Metabolism of amino acids and derivatives (C); S Phase (D), G1/S Transition (E), Cell Cycle, Mitotic (F) signaling pathway

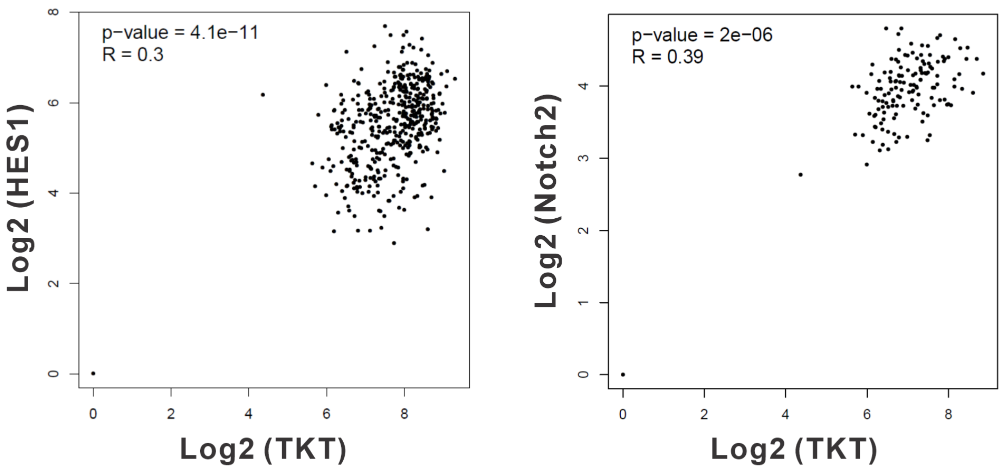

Supplementary Figure S5

Associations of TKT and HES1 or Notch

The expression of HES1 and Notch was positively correlated with TKT (HES1: R= 0.30; Notch2: R= 0.39).
